# The Endocannabinoid Reuptake Inhibitor WOBE437 Is Orally Bioavailable and Exerts Indirect Polypharmacological Effects via Different Endocannabinoid Receptors

**DOI:** 10.3389/fnmol.2018.00180

**Published:** 2018-05-28

**Authors:** Inés Reynoso-Moreno, Andrea Chicca, Mario E. Flores-Soto, Juan M. Viveros-Paredes, Jürg Gertsch

**Affiliations:** ^1^Institute of Biochemistry and Molecular Medicine, National Centre of Competence in Research TransCure, University of Bern, Bern, Switzerland; ^2^Laboratorio de Investigación y Desarrollo Farmacéutico, Departamento de Farmacología, Centro Universitario de Ciencias Exactas e Ingenierías, Universidad de Guadalajara, Guadalajara, Mexico; ^3^Laboratorio de Neurobiología Celular y Molecular, División de Neurociencias, Centro de Investigación Biomédica de Occidente, Instituto Mexicano del Segura Social, Guadalajara, Mexico

**Keywords:** allodynia, endocannabinoids, inflammation, monoarthritis, polypharmacology, bioavailability, endocannabinoid transport

## Abstract

Different anandamide (AEA) transport inhibitors show antinociceptive and antiinflammatory effects *in vivo*, but due to their concomitant inhibition of fatty acid amide hydrolase (FAAH) and overall poor bioavailability, they cannot be used unequivocally to study the particular role of endocannabinoid (EC) transport in pathophysiological conditions *in vivo*. Here, the potent and selective endocannabinoid reuptake inhibitor WOBE437, which inhibits AEA and 2-arachidonoylglycerol (2-AG) transport, was tested for its oral bioavailability to the brain. WOBE437 is assumed to locally increase EC levels in tissues in which facilitated EC reuptake intermediates subsequent hydrolysis. Given the marked polypharmacology of ECs, we hypothesized to see differential effects on distinct EC receptors in animal models of acute and chronic pain/inflammation. In C57BL6/J male mice, WOBE437 was orally bioavailable with an estimated *t*_max_ value of ≤20 min in plasma (C_max_ ∼ 2000 pmol/mL after 50 mg/kg, p.o.) and brain (C_max_ ∼ 500 pmol/g after 50 mg/kg, p.o.). WOBE437 was cleared from the brain after approximately 180 min. In addition, in BALB/c male mice, acute oral administration of WOBE437 (50 mg/kg) exhibited similar brain concentrations after 60 min and inhibited analgesia in the hot plate test in a cannabinoid CB1 receptor-dependent manner, without inducing catalepsy or affecting locomotion. WOBE437 significantly elevated AEA in the somatosensory cortex, while showing dose-dependent biphasic effects on 2-AG levels in plasma but no significant changes in *N*-acylethanolamines other than AEA in any of the tissues. In order to explore the presumed polypharmacology mediated via elevated EC levels, we tested this EC reuptake inhibitor in complete Freud’s adjuvant induced monoarthritis in BALB/c mice as a model of chronic inflammation. Repetitive doses of WOBE437 (10 mg/kg, i.p.) attenuated allodynia and edema via cannabinoid CB2, CB1, and PPARγ receptors. The allodynia inhibition of WOBE437 treatment for 3 days was fully reversed by antagonists of any of the receptors. In the single dose treatment the CB2 and TRPV1 antagonists significantly blocked the effect of WOBE437. Overall, our results show the broad utility of WOBE437 for animal experimentation for both p.o. and i.p. administrations. Furthermore, the data indicate the possible involvement of EC reuptake/transport in pathophysiological processes related to pain and inflammation.

## Introduction

The endocannabinoid system (ECS) is an endogenous pan-organ lipid signaling network that modulates numerous biological processes, including neurotransmission, metabolism and immune function (Maccarrone et al., 2015; Iannotti et al., 2016; Ligresti et al., 2016). The major endogenous agonists (i.e., endocannabinoids, ECs) for cannabinoid receptors CB1 and CB2 are the arachidonic acid (AA)-derived lipids 2-arachidonoyl glycerol (2-AG) and *N*-arachidonoylethanolamine (anandamide, AEA). However, these lipids also exert polypharmacological (i.e., pleiotropic) effects via additional proteins, including ion channels like transient receptor potential cation channel subfamily V member 1 (TRPV1) and peroxisome proliferator-activated receptor gamma (PPARγ), a nuclear receptor implicated in inflammatory processes (Di Marzo and De Petrocellis, 2012; Pertwee, 2015; Pistis and O’Sullivan, 2017; Villapol, 2018). Altered EC signaling in the brain has been associated with nociception (Piomelli and Sasso, 2014), learning and memory (Mechoulam and Parker, 2011), anxiety (Lisboa et al., 2017), among others. Overall, the ECS is involved in chronic inflammatory processes and related pain disorders (Lynch, 2016; Barrie and Manolios, 2017; Chiurchiù et al., 2018).

The indirect modulation of EC levels is an attractive therapeutic strategy as it may lead to fewer adverse effects than the direct activation of CB1 receptors in terms of neurotransmission, metabolism and immunomodulation (Di Marzo et al., 2004). In addition to directly targeting EC receptors, indirect cannabimimetics, e.g., inhibitors of the EC degrading enzymes fatty acid amide hydrolase (FAAH) or monoacylglycerol lipase (MAGL), are promising agents for the treatment of certain types of diseases, including inflammatory pain (Chicca et al., 2016). FAAH and MAGL inhibitors such as URB597 (Kathuria et al., 2003) and JZL184 (Long et al., 2009a), respectively, have been instrumental to elucidate the role of AEA and 2-AG in animal models of anxiety and depression (Busquets-Garcia et al., 2011; Bedse et al., 2014; Rubino et al., 2015; Zhang et al., 2015), but also inflammatory diseases and nociception (Trang, 2007; Woodhams et al., 2012, 2017).

Although AEA and 2-AG have different intracellular fates, they may share a common mechanism of membrane trafficking (i.e., facilitated cellular uptake) that is selective for ECs over arachidonate and other *N*-acylethanolamines (NAEs) (Fegley et al., 2004; Chicca et al., 2012; Nicolussi and Gertsch, 2015). While suitable inhibitors for *in vivo* research are available for most targets within the ECS (Blankman and Cravatt, 2013), the existing AEA uptake inhibitors lack potency and show poor selectivity over the other components of the ECS, in particular FAAH (Fowler, 2013; Nicolussi and Gertsch, 2015). This is potentially problematic for the interpretation of data obtained with such inhibitors in behavioral paradigms. Conversely, the novel EC transport inhibitor WOBE437 has been shown to selectively block EC cellular reuptake at low nanomolar concentrations in different cellular system *in vitro* and *in vivo*, without targeting CB receptors, FAAH, MAGL or fatty acid binding proteins (Chicca et al., 2017). Moreover, WOBE437 is currently the only EC transport inhibitor shown to penetrate the brain at bioactive concentrations (Chicca et al., 2017). Although its molecular mechanism of action remains unknown, evidence has been provided that it primarily acts via elevating EC levels in tissues where EC facilitated cellular reuptake plays a role, e.g., in the CNS or in immune cells (Chicca et al., 2017).

The majority of AEA transport inhibitors currently used exert a range of potential therapeutic effects in animal models (Ligresti et al., 2006; Khasabova et al., 2013; Murillo-Rodríguez et al., 2013; Batista et al., 2014). However, AEA uptake inhibitors like OMDM-2 (Ortar et al., 2003), UCM707 (López-Rodríguez et al., 2001), and AM404 (Beltramo et al., 1997) are poorly selective over FAAH and have not been tested for their oral bioavailability, which it is presumed to be low in animal experiments because some of these inhibitors are degraded by FAAH (Fegley et al., 2004; Chicca et al., 2017). The natural EC reuptake inhibitor guineensine (Nicolussi et al., 2014), despite being inactive at FAAH, has recently been shown to exert potent antiinflammatory effects *in vivo* but it also targets different CNS receptors *in vitro* which might explain its CB1 receptor-independent central effects (Reynoso-Moreno et al., 2017). Furthermore, few studies have addressed the uptake inhibition of 2-AG, which is the major endocannabinoid in the brain. 2-AG acts in concert with AEA, as evidenced from differential effects between selective and non-selective FAAH/MAGL inhibitors (Long et al., 2009b; Lau et al., 2014).

The aim of this study was to assess the oral bioavailability of WOBE437 to the brain and obtain data on its tissue distribution over time. We could correlate the oral dose of WOBE437 with both its pharmacological effect in acute pain and overall modulation of lipids related to the ECS. Here, we show that in chronic inflammation, the action of WOBE437 is mediated via different receptors, thus reflecting the pleiotropic action of ECs in complex pathophysiological conditions. These data indicate that the selective inhibition of EC reuptake could be a potential therapeutic strategy for chronic inflammatory conditions in which different receptors and signaling pathways cooperate in the etiopathology.

## Materials and Methods

### Animals

Male BALB/c or male C57BL6/J mice (8–10 weeks old; 20–25 g body weight) were either supplied by the Centro de Investigación Biomédica de Occidente or Jackson Laboratory and kept under standard environmental conditions (24 ± 2°C; light–dark cycle of 12:12 h) with food and water *ad libitum*. Mice were handled according to Mexican Federal Regulations for the Care and Use of Laboratory Animals NOM-062-ZOO-1999 (Mexican Ministry of Health) and according to the Swiss federal guidelines, which is in accordance with the Code of Ethics of the Directive 2010/63/EU.

### Oral Bioavailability Experiments

Male C57BL6/J or male BALB/c mice were orally administered by gavage with 10, 25, 50, or 100 mg/kg of WOBE437 (100 μL; olive oil and ethanol 8:2) using a metal feeding needle (20G, Kent Scientific, United states). The mice were sacrificed by decapitation after gavage administration. Brain, blood, liver, kidney, and spleen were collected for the LC-MS/MS analyses. All tissues were briefly washed with ice cold PBS and immediately snap-frozen and stored at -80°C. Whole brain samples were dissected between the hemispheres. Blood samples were immediately centrifuged to obtain plasma, which was stored at -80°C.

### Quantification of WOBE437 and Endocannabinoid Levels by LC-MS/MS

Samples extractions and LC-MS/MS measurements we performed as previously described (Chicca et al., 2017). Briefly, tissues were weighted and transferred in to extraction tubes containing three steel beads and 0.1 M formic acid. Tissues were homogenized and rapidly transferred to glass tubes containing 1.5 mL of ethyl acetate:hexane (9:1) 0.1% formic acid and internal standards (ISs). Samples were centrifuged and the organic phase was recovered. After evaporation, the extracts were reconstituted in 200 μL of ACN:ddH2O (8:2). 10 μL of the solution were injected in the LC-MS/MS system using the same LC and ionization protocols as described (Chicca et al., 2017).

### Microsomal Clearance

Human or mice microsome clearance experiment were carried out as according to a previously describe protocol (Soethoudt et al., 2017). Briefly, microsome preparation (0.5 mg/mL) plus cofactor NADPH solution was incubated with 1 μM of WOBE437 in 96-well plates at 37°C. After 1, 3, 6, 9, 15, 25, 35, and 45 min, 40 μL of the preparation was transferred and quenched with 3:1 (v/v) acetonitrile containing internal standards. Samples were then cooled and centrifuged before analysis by LC-MS/MS. Log peak area ratios (test compound peak area/internal standard peak area) are plotted against incubation time using a linear fit. The calculated slope is used to determine the intrinsic clearance. Data are obtained from single experiments measured with multiple time-points.

### Hot Plate Test

Acute pain was evaluated 1 h after gavage administration of WOBE437, at 10, 25, 50, and 100 mg/kg in BALB/c mice. The test was performed using a clean 54–56°C hot plate (Thermo Fisher Scientific, United states) with a Plexiglas cylinder. The latency to show the first nociceptive response (paw lick or foot shake) was measured. In order to evaluate CB1 receptor antagonism, 5 mg/kg of rimonabant (Pharmaserv AG, Switzerland) was injected intraperitoneally (i.p.) (20 μL, in DMSO) 30 min before WOBE437 administration.

### Tetrad Test

The tetrad test was performed in the following order: rectal temperature, catalepsy, locomotion and analgesia. Rectal temperature was measured before (basal) and 1 h post gavage administration by using a thermocouple probe (Physitemp Instruments Inc., United states) and results were reported as the difference (Δ) between both temperatures. The bar test was used to evaluated catalepsy by placing the mouse in an imposed position with both forelimbs resting on a bar of 4 cm high; the end point of catalepsy was considered when both front limbs were removed or remained over 120 s. Locomotor activity was determined by placing the mouse on a rotarod (Erweka, Germany) at 4 rpm, the latency to fall was measured with a cut-off time of 120 s. Each mouse was previously trained to walk over the rotarod for at least 120 s. Catalepsy and locomotion were measured in three trials. Analgesia was evaluated with the hot plate test, each mouse was placed in a 54–56°C hot plate (Thermo Fisher Scientific, United states) with a Plexiglas cylinder, the latency to first nociceptive response (paw lick or foot shake) was measured.

### Mouse Model of Complete Freund’s Adjuvant Induced Monoarthritis

BALB/c mice were anesthetized with xylazine/ketamine (5 and 10 mg/kg, respectively) and subsequently monoarthritis was induced by intra-articular injection of complete Freund’s adjuvant (CFA, 40 μL; Sigma-Aldrich, St. Louis, MO, United states) into the right knee joint. The inflammation was allowed to develop for 14 days prior to pharmacological treatments starting at day 15 for 1 or 3 days, respectively. Mechanical sensitivity (allodynia) and knee diameter (edema) were evaluated 1 h post treatment in the ipsilateral and contralateral knees. Articular sensitivity was evaluated using a digital algometer (Bioseb, United states). The knee withdrawal threshold was determined by applying slow increments of force into the joint until the mice showed a signal of pain (withdrawal reflex or vocalization). A cut-off force of 300 g was considered to avoid damage in the tissue. The measurements were done in triplicates and the average was considered for the statistical analysis. To evaluate the degree of inflammation, knee diameter was measured in both extremities using a digital micrometer (Mitutoyo, United states). WOBE437 was administered at doses of 2.5, 5, or 10 mg/kg, either as single dose or for 3 days treatment. To evaluate the involvement of CB1 and CB2 receptors, TRPV1 and PPARγ, rimonabant (5 mg/kg, CB1 receptor antagonist/inverse agonist), SR144528 (3 mg/kg, CB2 receptor antagonist/inverse agonist; Cayman Chemical, United states), capsazepine (5 mg/kg, TRPV1 antagonist; Tocris Bioscience, United Kingdom) and GW9662 (3 mg/kg, PPARγ antagonist; Tocris Bioscience, United Kingdom) were injected 30 min before WOBE437 administration. Indomethacin (5 mg/kg; Sigma-Aldrich, United states) was used as a reference drug (positive control). For all the compounds, dimethylsulfoxide (DMSO) was used as vehicle (20 μL, i.p.).

### Open Field Test

Potential motor changes were evaluated in an open field box (40 cm × 40 cm × 30 cm, Plexiglas). The mice were individually placed in the center and allowed to move freely for 5 min. The locomotion activity was recorded with the OpenFieldTest^©^ (Reynoso-Moreno et al., 2017) and traveled distance was evaluated automatically. After every experiment, the box was cleaned with 70% ethanol to remove odors.

### Real-Time PCR

To evaluate gene expression of CB receptors and the main enzymes involve in AEA and 2-AG biosynthesis, somatosensory cortex, thalamus and knee articular tissue were recover after 3 days of treatment with vehicle or WOBE437 in the complete Freund’s adjuvant induced monoarthritis model. Total RNA extraction and reverse transcription were carried out with a previously described protocol (Castro-Torres et al., 2015). The kit LightCycler^®^ FastStart DNA Master^PLUS^ SYBR Green I (Roche, United states) was used for the real-time PCR, according to manufacturer protocol, and the reaction was performed in the LightCycler 2.0 equipment (Roche, United states). The relative gene expression was calculated by using the method known as 2ˆ(-ΔΔCt) (Livak and Schmittgen, 2001). ΔΔCt was calculated using the following formula:

(1) $$\Delta \Delta \mathrm{Ct}=({\mathrm{Ct}}_{\mathrm{TE}}-{\mathrm{Ct}}_{\mathrm{HE}})-({\mathrm{Ct}}_{\mathrm{TC}}-{\mathrm{Ct}}_{\mathrm{HC}})$$

Ct_TE_: *Ct* for the tested gene (*Cnr1, Cnr2, Nape-pld, Dagla*) in the experimental or control group

Ct_HE_: Ct for the housekeeping gene (*Actb*) in the experimental or control group

Ct_TC_: mean Ct for the tested gene in the control group

Ct_HC_: mean Ct for the housekeeping gene in the control group

Finally 2ˆ(-ΔΔCt) was calculated to obtain the fold change of gene expression. Every sample (*n* = 6-15, per group and region) was analyzed in duplicated and the mean value was considered for ΔΔCt calculation. Beta-actin was used as the housekeeping gene and mean of vehicle group was used as a calibrator. Sequences and size products of the primers for beta actin (*Actb*) cannabinoid CB1 receptor (*Cnr1*), cannabinoid CB2 receptor (*Cnr2*), diacylglycerol lipase alpha (*Dagla*), *N*-acylphosphatidylethanolamine specific phospholipase D (*Nape-pld*) are shown in **Table 1**.

**Table 1:** Sequences of primers used in the real-time PCR.

Gen		Primers	Product size (bp)	GenBank access number
*Cnr1*	Sense	5′-CTT GCA GAT ACC ACC TTC CGT-3′	142	NM_007726.3
	Antisense	5′-CCC TGA AGG AAG TTA GAG GGA-3′		
*Cnr2*	Sense	5′-GAC AGA AGT GAC CAA CGG CT-3′	76	X93168.1
	Antisense	5′-GCC ACT GCT CAG GAT CAT GT-3′		
*Napepld*	Sense	5′-GCG CCA GAA TTC AGT GCA GA-3′	175	NM_178728.5
	Antisense	5′-GAG CAC ATT CGG GAT GGA GA-3′		
*Dagla*	Sense	5′-CTC GTC CTG CCA GCT ATC TT-3′	157	NM_198114.2
	Antisense	5′-TAC AGC TCA GAA GGA TGC CC-3′		
*Actb*	Sense	5′-GGC CAA CCG TGA AAA GAT GA-3′	77	NM_007393.5
	Antisense	5′-CAG CCT GGA TGG CTA CGT ACA-3′		

*Actb, beta actin; bp, base of pairs; Cnr1, cannabinoid CB1 receptor; Cnr2, cannabinoid CB2 receptor; dagla, diacylglycerol lipase alpha; Napepld, N-acylphosphatidylethanolamine specific phospholipase D (Nape-pld).*

### Statistical Analysis

All data are presented as mean values ± SD and were analyzed by non-parametrical methods using a Kruskal–Wallis test followed by Mann–Whitney U as a *post hoc* test. A confidence level of *p* < 0.05 was considered statistically significant. Analyses were carried out using the GraphPad Prism software version v5.0 (La Jolla, CA, United States).

## Results

### The Endocannabinoid Reuptake Inhibitor WOBE437 Is Orally Bioavailable

**FIGURE 1:**
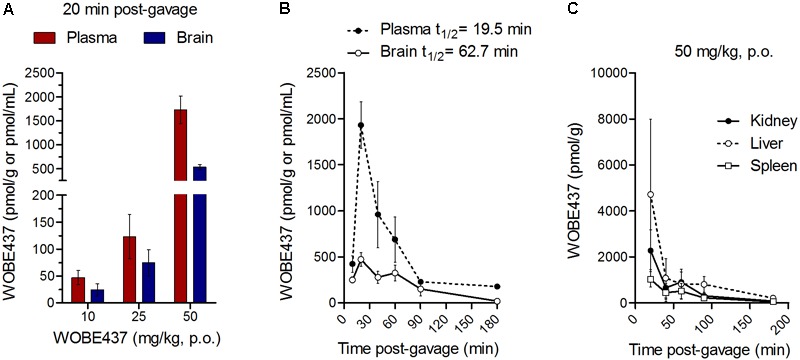
WOBE437 is rapidly biodistributed after oral administration in C57BL6/J male mice. **(A)** After 20 min post-gavage, WOBE437 was dose-dependently absorbed and reached quantifiable concentrations in brain and plasma. Data show mean values ± SD of 5–10 mice. **(B)** Time course of WOBE437 concentration in brain and plasma showing an estimated T_max_ of 10–20 min after administration. Data show mean values ± SD of 5–10 mice. **(C)** Time course of WOBE437 concentrations in kidney, liver, and spleen, showing the highest concentrations in liver 20 min after administration. p.o., per os. Data show mean values ± SD of 5–10 mice.

Oral administration of WOBE437 in male C57BL6/J mice showed a complete biodistribution after 20 min, with corresponding brain levels of 24.7 ± 25.3 pmol/g using a dose of 10 mg/kg and 534.5 ± 109.9 pmol/g using a dose of 50 mg/kg (**Figure 1A**). In plasma, WOBE437 reached 47.3 ± 32.5 and 1731.5 ± 703.4 pmol/mL after oral doses of 10 or 50 mg/kg, respectively (**Figure 1A**). In order to characterize the tissue distribution of WOBE437 over time, brain and plasma samples were recovered at different time-points (10, 20, 40, 60, 90, and 180 min) after gavage administration of 50 mg/kg. The highest concentration of WOBE437 was found at ≤20 min (*t*_max_), reaching apparent *C*_max_ values of 471.7 ± 182.6 pmol/g in brain and 1931 ± 564 pmol/mL in plasma, respectively (**Figure 1B**). The apparent volume of distribution (Vd) at *C*_max_ was 80.4 L/kg and at the steady state 609 L/kg, thus large and reflecting lipid solubility and massive tissue penetration/retention. In brain, the value obtained with 50 mg/kg, p.o. is equivalent to an estimated concentration of 399.7 ± 154.7 nM. WOBE437 is cleared from the brain after approximately 180 min. Furthermore, in kidney, liver, and spleen, the *t*_max_ values of WOBE437 were ≤20 min after administration (**Figure 1C**). The highest concentration was found in liver with 4720 ± 3273 pmol/g, followed by kidney (2277 ± 916 pmol/g) and spleen (1030 ± 339 pmol/g). Between 20 and 40 min, the hepatic concentration of WOBE437 showed the most significant reduction, dropping by a factor 4 from 4720 to 1080 pmol/g (**Figure 1C**). In preliminary experiments with human and mouse liver microsomes the clearance of WOBE437 was estimated as 657 and 174 μL/min/kg, respectively (**Table 2**). These values suggested an estimated maximal bioavailability of 17% in mice and 4% in humans.

**Table 2:** Clearance of WOBE437 calculated after incubation for 2 h with human and mouse liver microsomes.

	Mouse	Human
Clearance	174 μL/min/kg	657 μL/min/kg
MAB	17%	4%

*The maximal achievable bioavailability (MAB) was estimated from the clearance*.

### Nociceptive Effect After Single Oral Dose of WOBE437

**FIGURE 2:**
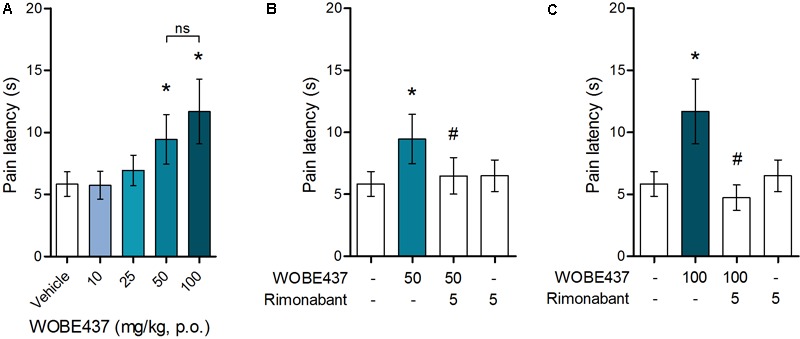
Oral administration of WOBE437 attenuates acute pain in the hot plate test in BALB/c male mice in a CB1 receptor-dependent manner. **(A)** Dose-response curve of WOBE437 in the pain latency response in the hot plate test. 50 mg/kg of WOBE437 p.o. was the minimum dose to significantly increase pain threshold. The analgesic effect of **(B)** 50 mg/kg and **(C)** 100 mg/kg of WOBE437 was completely abolished by pre-treatment with rimonabant (5 mg/kg, i.p.). All doses are expressed in mg/kg. Rimonabant was injected i.p. 30 min before gavage administration of WOBE437. Data show mean values ± SD of 5–10 mice. Data were compared using Kruskal–Wallis test followed by Mann–Whitney test. ^∗^*p* < 0.05 vs. vehicle; ^#^*p* < 0.05 vs. WOBE437; p.o. per os; ns, no significant.

**FIGURE 3:**
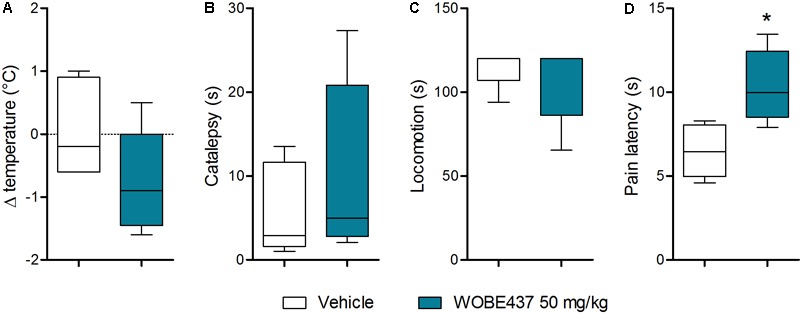
Oral administration of 50 mg/kg WOBE437 did not elicit all the effects in the tetrad test in BALB/c male mice. **(A)** Change in body temperature **(B)** latency of catalepsy, **(C)** locomotion, and **(D)** latency of pain response 1 h after gavage administration of vehicle or 50 mg/kg of WOBE437. Data show median percentile 25, percentile 75 minimum and maximum of five mice. Data were compared using Mann–Whitney test. ^∗^*p* < 0.05 vs. vehicle.

**FIGURE 4:**
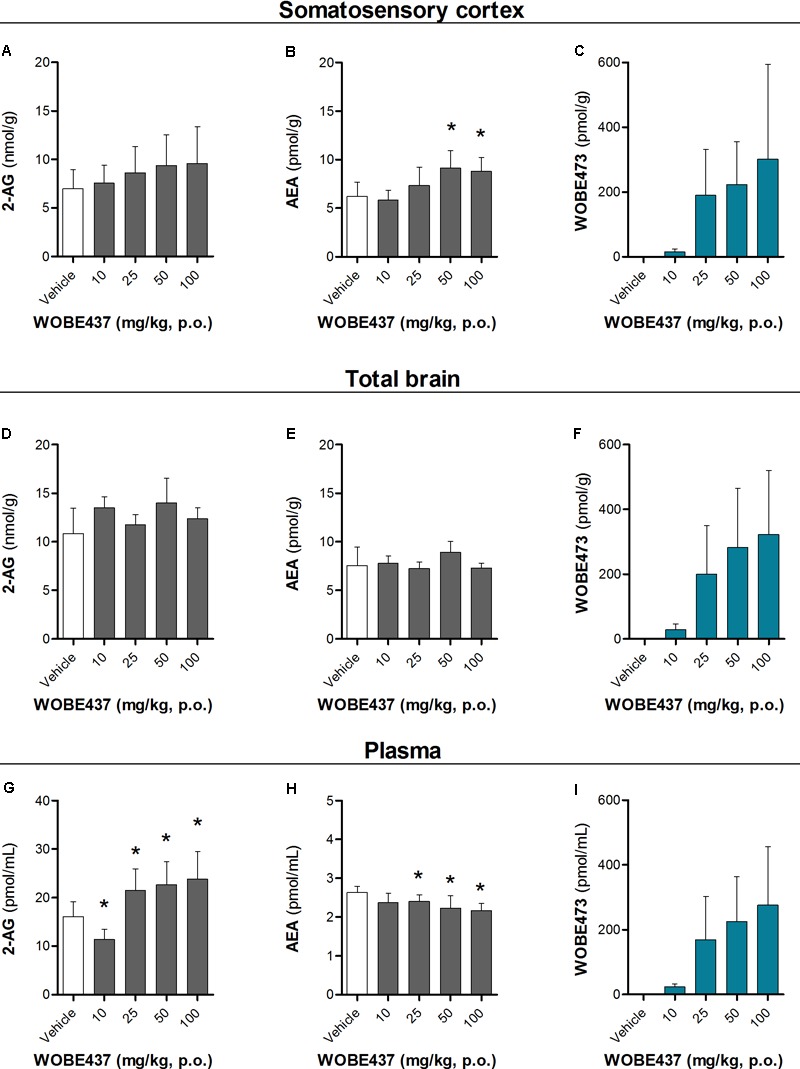
Changes in endocannabinoid levels 1 h after oral administration of WOBE437 in BALB/c male mice. In somatosensory cortex, WOBE437 did not change **(A)** 2-AG levels but significantly increased **(B)** AEA levels with a single 50 mg/kg dose. **(C)** Concentration of WOBE437 in somatosensory cortex. In total brain homogenate, **(D)** 2-AG and **(E)** AEA did not significantly change after oral administration of a single dose of 50 mg/kg of WOBE437. **(F)** Concentration of WOBE437 in total brain homogenate. **(G)** 2-AG levels were significantly increase in plasma with a slightly decrease in **(H)** AEA. **(I)** Concentration of WOBE437 in plasma. All data show mean values ± SD of at least 7 to 10 mice. Groups were compared using Kruskal–Wallis test followed by Mann–Whitney test. ^∗^*p* < 0.05 vs. vehicle. 2-AG, 2-arachidonoylglycerol; AEA, anadamide; p.o. per os.

**FIGURE 5:**
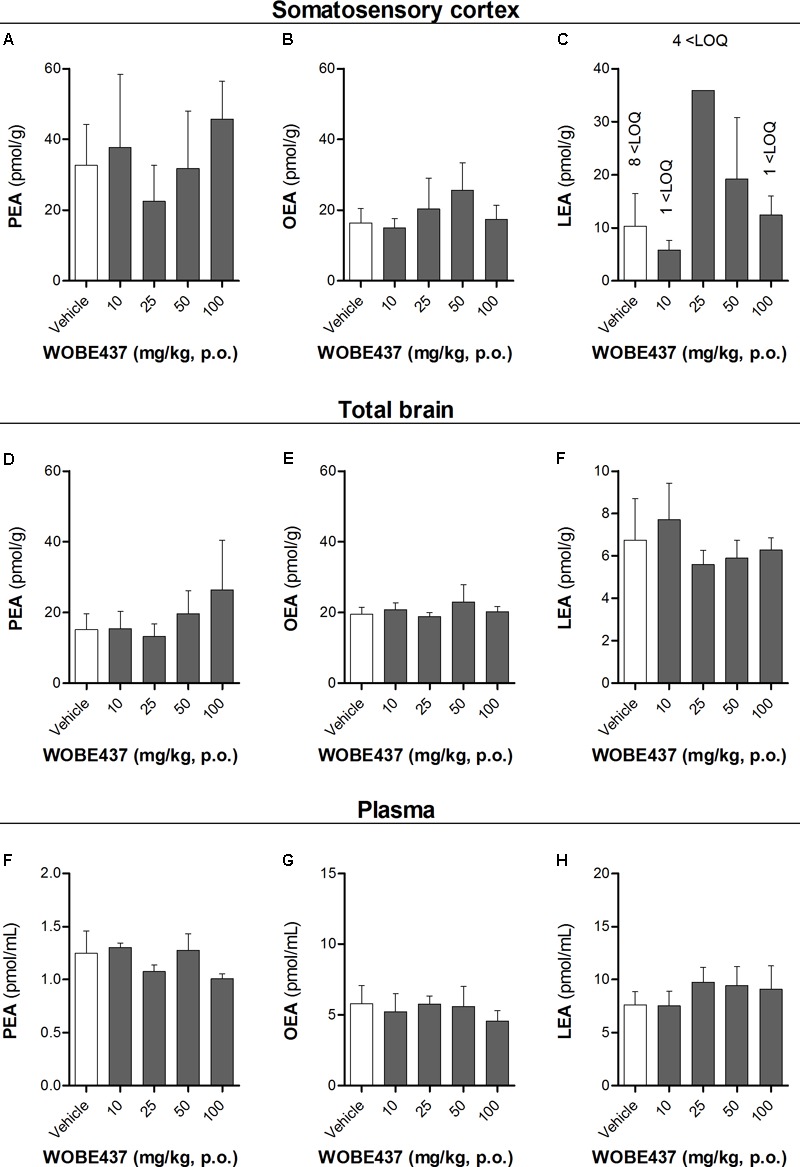
Levels of *N*-acylethanolamines 1 h after oral administration of WOBE437 in BALB/c male mice. **(A)** Palmitoylethanolamide (PEA), **(B)** oleoylethanolamine (OEA) and **(C)** linoleoylethanolamide (LEA) levels in somatosensory cortex. **(D)** PEA, **(E)** OEA, and **(F)** LEA levels in total brain homogenate. **(G)** PEA, **(H)** OEA, and **(I)** LEA levels in plasma. All data show mean values ± SD of at least 5 to 10 mice. Groups were compared using Kruskal–Wallis test followed by Mann–Whitney test. ^∗^*p* < 0.05 vs. vehicle. LOQ, limit of quantification; p.o. per os.

Since WOBE437 has previously shown analgesic effects in different models of acute pain after single i.p. injection (Chicca et al., 2017), we evaluated the pharmacological effects of WOBE437 after oral administration in BALB/c male mice. In a dose-response curve, 50 mg/kg, p.o. of WOBE437 was the minimum dose necessary to significantly increase the pain threshold from 5.8 ± 0.9 s in the vehicle group to 9.4 ± 1.9 s (61.7% increase, **Figure 2A**). WOBE437 at 100 mg/kg, p.o. marginally further increased the pain threshold to 11.7 ± 2.6 s, thus not significantly more than 50 mg/kg, with an estimated effective dose (ED50) value of 42.5 ± 3.9 mg/kg. The analgesic effect of WOBE437 was mediated through the CB1 receptor as the antinociceptive effect was completely abolished after rimonabant pre-treatment (**Figures 2B,C**). Additionally, the oral administration of 50 mg/kg of WOBE437 was evaluated in the tetrad test (**Figure 3**). No statistical differences were found in body temperature, catalepsy or motor coordination, but significant antinociception was measured. However, a tendency to induce hypothermia (**Figure 3A**) and catalepsy (**Figure 3B**) was observed as the body temperature decreased from +0.1 ± 0.8°C (control) to -0.8 ± 0.8°C (WOBE437 50 mg/kg) and the latency of catalepsy increased from 5.9 ± 5.4 s (control) to 10.5 ± 10.6 s (WOBE437 50 mg/kg). To further characterize the effects of oral administration of WOBE437, brain and plasma samples were collected after the hot plate test to analyze the EC levels. In somatosensory cortex, 50 mg/kg of WOBE437 showed a significant increase in AEA levels (**Figure 4B**) but no changes in 2-AG (**Figure 4A**). In total brain, WOBE437 showed a tendency to increase both AEA and 2-AG levels (**Figures 4D,E**). Interestingly, in plasma we measured a dose-dependent biphasic effect on 2-AG with a significant rise in 2-AG levels after 50 and 100 mg/kg of WOBE437 (**Figure 4G**). AEA showed a statistically significant but slight reduction in plasma (**Figure 4H**). The levels of WOBE437 were similar in somatosensory cortex, total brain and plasma (**Figures 4C,F,I**) and in the same range as in male C57BL6/J mice (**Figure 1B**), despite BALB/c showing a tendency toward lower plasma levels (not statistically significant). Overall, the oral administration resulted in high variability of WOBE437 tissue concentrations. No changes in the levels of *N*-acylethanolamines other than AEA were measured in the somatosensory cortex, total brain or plasma (**Figure 5**).

### WOBE437 Indirectly Triggers Polypharmacological Effects in a Model of Chronic Inflammation

**FIGURE 6:**
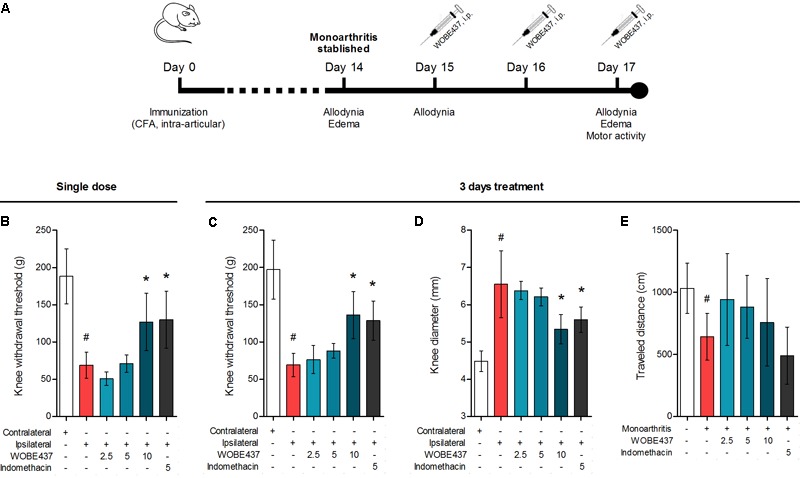
Effect of WOBE437 on allodynia and inflammation after a single dose or 3 days treatment in a mouse model of monoarthritis. **(A)** Treatment scheme of monoarthritis induced by knee immunization with complete Freund’s adjuvant (CFA) (40 μL, intra-articular) in which the inflammatory process was allowed to develop for 14 days. Intraperitoneal treatments with WOBE437 were carried on days 15 to 17. **(B)** Allodynia was evaluated upon single dose treatments in which 10 mg/kg of WOBE437 increased the pain threshold. After 3 days of treatment, 10 mg/kg of WOBE437 improved **(C)** allodynia and reduced **(D)** edema. **(E)** The development of monoarthritis significantly decreased the travel distance in the open field test, but no significant changes were observed after WOBE437 treatment due to high variability. Indomethacin was used as a reference drug. All doses are shown in mg/kg, i.p. Allodynia was evaluated by mechanical sensitivity and edema through knee diameter and both were measured 1 h after pharmacological treatments. All data show mean values ± SD of at least 6 to 15 mice. Groups were compared using Kruskal–Wallis test followed by Mann–Whitney test. ^∗^*p* < 0.05 vs. ipsilateral/vehicle; ^#^*p* < 0.05 vs. contralateral/healthy; i.p., intraperitoneally.

Considering our previous data showing the analgesic and antiinflammatory effects of WOBE437 after single i.p. injection (Chicca et al., 2017) and the confirmation of CB1 receptor-dependent antinociception after oral WOBE437 administration (**Figure 2**), we decided to further evaluate its pharmacological properties and underlying mechanism(s) in a chronic model of inflammatory pain. After the induction of monoarthritis by intra-articular injection of CFA in mice (**Figure 6A**), a single dose of WOBE437 at 10 mg/kg, i.p., was able to significantly decrease allodynia (**Figure 6B**). After 3 days treatment, allodynia was reduced by increasing the pain threshold from 69.0 ± 15.7 g in the vehicle group to 136. 3 ± 31.7 g in the group treated with WOBE437 10 mg/kg, i.p., reflecting a reduction of 52% in allodynia as compared to the contralateral knee (197.0 ± 39.6 g) (**Figure 6C**). Furthermore, the inflammation (edema) was reduced by 58% resulting in a decrease of the knee diameter from 6.5 ± 0.9 mm in the vehicle group to 5.3 ± 0.4 mm in the WOBE437 10 mg/kg group as compared to the contralateral knee (4.5 ± 0.3 mm) (**Figure 6D**). The doses of 2.5 and 5.0 mg/kg, i.p., did not show any significant changes. The spontaneous motor activity was measured, but without significant observable changes upon WOBE437 treatment. Nevertheless, a noteworthy tendency to improve spontaneous locomotion was noticed in the monoarthritic group treated with WOBE437 2.5 mg/kg (**Figure 6E**).

**FIGURE 7:**
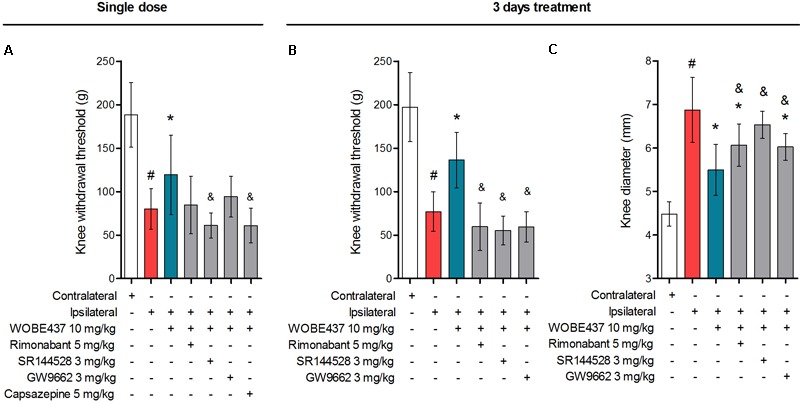
Polypharmacological effects observed after 3 days treatment with 10 mg/kg of WOBE437. **(A)** The antiallodynia effects of 10 mg/kg of WOBE437 after single dose were significantly reversed by the CB2 receptor antagonist SR144528 and the TRPV1 antagonist capsazepine. In the 3 days treatment scheme, **(B)** antiallodynia and **(C)** the antiinflammatory effects were prevented by antagonists of the CB1 receptor (rimonabant), CB2 receptor (SR144528) and PPARγ (GW9662). Rimonabant, SR144528 or GW9662 were administered i.p. 30 min before WOBE437. Allodynia was evaluated by mechanical sensitivity and edema measuring knee diameter; both were assessed 1 h after WOBE437 treatment. All compounds were administered i.p. Groups were compared using Kruskal–Wallis test followed by Mann–Whitney test. ^∗^*p* < 0.05 vs. ipsilateral/vehicle; ^#^*p* < 0.05 vs. contralateral/vehicle; ^&^*p* < 0.05 vs. WOBE437; i.p., intraperitoneally.

**FIGURE 8:**
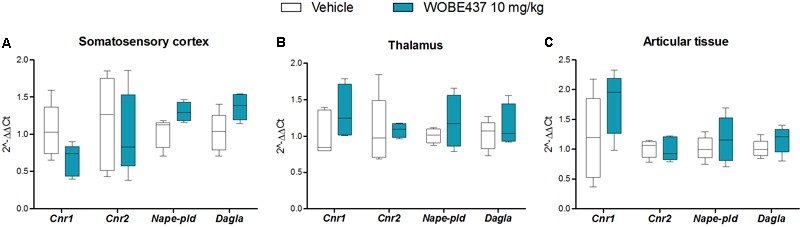
Changes in total RNA levels of EC components after the CFA-induced monoarthritis model and 3 days treatment with WOBE437 10 mg/kg, i.p., in BALB/c mice. Total RNA levels of cannabinoid CB1 receptor (*Cnr1*), cannabinoid CB2 receptor (*Cnr2*) *N*-acylphosphatidylethanolamine specific phospholipase D (*Nape-pld*) and diacylglycerol lipase (*Dagla*) did not show any significant changes in **(A)** somatosensory cortex, **(B)** thalamus, or **(C)** articular tissue. Beta-actin was used as housekeeping gene. mRNA levels were determined by RT-PCR. All data show median, percentile 75, percentile 25, min. and max. of at least 6 to 15 mice. Groups were compared using Kruskal–Wallis test followed by Mann–Whitney test. ^∗^*p* < 0.05 vs. ipsilateral/vehicle.

In an attempt to better characterize the downstream mechanism(s) involved in the pharmacological effects mediated by WOBE437 in the monoarthritis model, we pre-treated the mice with cannabinoid CB1 receptor, cannabinoid CB2 receptor, PPARγ and TRPV1 antagonists. In the single dose experiment, the antiallodynia effects of WOBE437 (10 mg/kg, i.p.) were clearly mediated by cannabinoid CB2 and TRPV1 receptors because the CB2 receptor antagonist/inverse agonist SR144528 (3 mg/kg, i.p.) and the TRPV1 antagonist capsazepine (5 mg/kg, i.p.) completely prevented the improvement in mechanical sensitivity by WOBE437 (**Figure 7A**). Conversely, the CB1 antagonist/inverse agonist rimonabant and PPARγ antagonist GW9662 did not block the effect of WOBE437 in this experiment. Interestingly, a clear multi-target mechanism was seen after 3 days treatment with WOBE437 10 mg/kg, i.p., because the antiallodynia and antiinflammatory effects were fully abolished by each of the cannabinoid CB1 receptor antagonist/inverse agonist rimonabant (5 mg/kg, i.p.), the CB2 antagonist SR144528 (3 mg/kg, i.p.) and PPARγ antagonist GW9662 (3 mg/kg, i.p.) (**Figures 7B,C**). On the other hand, the antiinflammatory effect was only partially prevented with rimonabant and GW9662 (**Figure 7C**), indicating additive contributions of CB1 and PPARγ receptors, respectively. In the 3 days WOBE437 treatment we could not use the TRPV1 antagonist capsazepine because the mice showed an unexpected hypersensitivity. In the monoarthritic mice, WOBE437 treatment did not lead to any significant change in the expression of the cannabinoid CB1 receptor (*Cnr1*), the cannabinoid CB2 receptor (*Cnr2*) or the enzymes involved in EC production, namely diacylglycerol lipase (*Dagla*) and N-acylphosphatidylethanolamine specific phospholipase D (*Nape-pld*) (**Figure 8**), thus excluding broad transcriptional effects on the ECS.

## Discussion

### WOBE437 Is Orally Bioavailable to the Brain and Exerts Cannabinoid CB1 Receptor-Dependent Antinociceptive Effects

As outlined by Arrowsmith et al. (2015), chemical probes are powerful tools with increasing impact on biomedical research. However, probes and inhibitors of poor quality or that are used incorrectly generate misleading results. Among the AEA transport inhibitors, this problem is inherent because they do not generally fulfill the criteria of a good chemical probe, such as potency, selectivity and bioavailability. Importantly, none of the AEA/EC transporter inhibitors (UCM707, OMDM-2, AM404, guineensine) has been investigated for general bioavailability, pharmacokinetic parameters or brain penetration by analytical methods (see below). We recently reported the development of WOBE437 as a novel highly potent and selective EC reuptake inhibitor (SERI) which specifically increases/modulates the levels of the two main ECs AEA and 2-AG *in vitro* and *in vivo* (Chicca et al., 2017). WOBE437 showed relevant pharmacological effects in different animal models of acute pain, anxiety and endotoxemia, reaching bioactive concentrations in the brain after intraperitoneal injection (Chicca et al., 2017). Here, we assessed the basic oral bioavailability of WOBE437, its distribution to the brain, plasma and peripheral tissues and evaluated its corresponding antinociceptive and cannabimimetic effects. Considering the stress induced by gavage feeding, the hot plate assay was an ideal nociception paradigm as it avoids chronic stress, unlike, e.g., chronic inflammation. Our results showed quantifiable levels of WOBE437 in brain already 10 min after oral gavage, reaching the highest concentration 20 min after administration, with an estimated *t*_max_ of ≤20 min in plasma (*C*_max_ ∼2000 pmol/mL after 50 mg/kg, p.o.) and brain (*C*_max_ ∼500 pmol/g after 50 mg/kg, p.o.). The WOBE437 concentration in brain remained relatively stable between 300 and 400 pmol/g up to 1 h and was still detectable 3 h after administration (18 pmol/g). A control over the bioavailability and basic pharmacokinetics requirements (e.g., reaching bioactive tissue concentrations) are fundamental to understand the pharmacological effects measured in behavioral tests. However, the oral bioavailability of the currently used non-selective AEA transport inhibitors remains unknown. We and others have previously shown that AEA uptake inhibitors like UCM707, AM404, and VDM11 are substrates for h/rFAAH or hMAGL, which strongly suggest their metabolic instability, i.e., degradation after systemic administration (Fegley et al., 2004; Vandevoorde and Fowler, 2005; Chicca et al., 2017). Conversely, WOBE437 is not affected by the hydrolytic activity of FAAH and MAGL and is not modified by COX-2 (Chicca et al., 2017). Nevertheless, the present data show that WOBE437 is significantly metabolized by liver microsomes (human and mouse) after 2 h of incubation, resulting in an estimated oral bioavailability of 4% (human) and 17% (mouse). In the hot plate test, WOBE437 exerted significant analgesic effects at the dose of 50 mg/kg upon oral administration, while the fully effective dose was 10 mg/kg upon systemic administration (i.p.) (Chicca et al., 2017). This difference suggests an overall 20% bioavailability compared to the i.p. administration route, in agreement the estimated maximal bioavailability of 17% calculated in mouse liver microsomes. According to these findings, one could assume that 50 mg/kg, p.o. is almost equivalent to 10 mg/kg, i.p. (i.e., a factor 5). However, the brain levels were approximately 10 times lower after p.o. as compared to i.p. Our previous findings showed that 10 mg/kg of WOBE437 i.p. induced a slight but significant effect in the entire tetrad test battery (hypothermia, catalepsy, hypolocomotion, and analgesia) (Chicca et al., 2017). Therefore, we also tested the effects of 50 mg/kg of WOBE437 p.o. in BALB/c mice. However, statistical significance was only found for analgesia 1 h after oral administration of WOBE437 but only a tendency to induce hypothermia and catalepsy. This can be explained by the lower levels of WOBE437 in the brain.

One hour after oral administration of WOBE437 at the single dose of 50 mg/kg, a significant increase of AEA levels in the somatosensory cortex was measured, with only a tendency to increase AEA in the total brain. These findings point to a brain region-specific increment in the AEA levels, in agreement with the different EC levels across brain regions and changes in a tissue specific manner (González et al., 2002; Bystrowska et al., 2014; Chicca et al., 2017). Moreover, 2-AG concentration showed a tendency to increase in total brain, from 10.8 ± 2.6 nmol/g to 13.9 ± 2.6 nmol/g after 50 mg/kg of WOBE437, without significant changes in somatosensory cortex. This lack of statistical significance in somatosensory cortex might be due to limited involvement of 2-AG in this area under basal conditions, because significant increase in 2-AG levels have been shown after stress or chronic constriction injury in dorsal midbrain and particularly in periaqueductal grey matter (PAG) (Hohmann et al., 2005; Petrosino et al., 2007). On the contrary, the treatment with 25, 50, and 100 mg/kg of WOBE437 induced a significant increase by 40% of 2-AG levels from 16.1 ± 2.9 pmol/mL to 21.4 ± 4.5 pmol/mL (25 mg/kg), 22.6 ± 4.8 pmol/mL (50 mg/kg) and 23.8 ± 5.7 pmol/mL (100 mg/kg) in plasma. On the other hand, the concentration of AEA in plasma was weakly but significantly reduced by 23%, from 2.6 ± 0.2 pmol/mL in the control group to 2.2 ± 0.3 pmol/mL in the group treated with WOBE437 at 50 mg/kg. This slight decrease in AEA levels may not cause biological effects but could rather reflect differential AEA transport kinetics between AEA and 2-AG in a complex tissue environment. We have previously shown that AEA and 2-AG compete for cellular uptake *in vitro* with different affinities (Chicca et al., 2012). Finally, the effects of WOBE437 on circulating ECs upon i.p. administration exhibited yet a different pattern leading to significant increase of 2-AG levels as compared to vehicle (after 15 min) but without affecting AEA levels (Chicca et al., 2017).

### Selective Inhibition of Endocannabinoid Uptake Through WOBE437 Triggers Polypharmacological Effects in Inflammatory Pain

To evaluate the preclinical potential of WOBE437, or in general of selective inhibition of the EC reuptake process, chronic inflammatory pain was induced by the intra-articular injection of CFA, with the subsequent development of monoarthritis-like conditions. Consecutive treatment for 3 days with 10 mg/kg of WOBE437 significantly attenuated allodynia and edema induced during monoarthritis. The allodynia was already reduced by single injection of 10 mg/kg of WOBE437. In models of CFA-induced persistent inflammatory pain, the antiallodynia effects of cannabinomimetics such as WIN55212-2, AM404, and ajulemic acid, have been previously shown to be mediated either via cannabinoid CB1 receptors or cannabinoid CB2 receptors (Mitchell et al., 2007; Vann et al., 2007; Li et al., 2017). Moreover, both cannabinoid CB1 and CB2 receptors have shown to be involved in the antiallodynic effect of JZL195, a dual inhibitor of AEA and 2-AG degradation (Anderson et al., 2014), suggesting a pleotropic multi-target mechanism associated to the simultaneous increase of both AEA and 2-AG levels (Di Marzo and De Petrocellis, 2012; Pertwee, 2015; Pistis and O’Sullivan, 2017). Given the fact that WOBE437 inhibits the reuptake of both AEA and 2-AG, we wondered whether the pharmacological action resembles the one observed with JZL195, but with a different mechanism of action. Interestingly, the selective and independent blockage of either, CB1 receptor, CB2 receptor or PPARγ fully reversed the antiallodynic effect induced by the treatment with WOBE437 for 3 days. Similarly, the antiinflammatory effect of WOBE437 (3 days of treatment), measured by the reduction of the knee diameter (edema), was partially and independently reversed by all three receptor antagonists. However, in the single dose treatment with WOBE437, only the selective CB2 receptor antagonist SR144528 and the TRPV1 antagonist capsazepine were able to fully block the antiallodynic effect of WOBE437, while selective antagonists of CB1 receptor and PPARγ did not achieve a significant reduction. Because AEA is the endogenous TRPV1 agonist (Smart et al., 2000) and capsazepine was able to block the effect of WOBE437 in the single dose treatment we assume that the increase of AEA levels induced by WOBE437 in the somatosensory cortex caused the underlying pharmacology. Analgesic effects of TRPV1 agonist have been reported after central activation of TRPV1 receptor channels within the descending antinociceptive pathway (Starowicz et al., 2007a; Mascarenhas et al., 2015), which includes spinal and supraspinal structures, such as PAG and rostral ventromedial medulla (RVM). In addition, intrathecal or intra-PAG administration of AEA have shown to induce antinociceptive or antiallodynia effects through TRPV1 activation (Maione et al., 2006; Horvath et al., 2008; Starowicz et al., 2012). In the CFA monoarthritis model, TRPV1 have shown to mediate analgesic effects by integrating different stimuli (Szabó et al., 2005). In addition, the expression of TRPV1 in cortical neurons and their functional role in modulating synaptic activity was recently described in mice suffering from neuropathic pain (Marrone et al., 2017). Furthermore, it has been propose that intra-PAG administration of capsaicin induce an antinociceptive response involving the activation of presynaptic TRPV1 receptors in glutamatergic neurons, activation of postsynaptic mGlu5 receptors, release of 2-AG via diacylglycerol lipase activation, activation of presynaptic CB1 receptor and inhibition of GABA release, which leads to disinhibition of the descending pain inhibitory pathway (Liao et al., 2011). On the other hand, it has also been reported that high concentrations of AEA at the peripheral level leads to nociception or hyperalgesia via TRPV1 receptors on primary sensory neurons, while low concentrations leads to antinociception via CB1 receptor (Gauldie et al., 2001; Morisset et al., 2001; Starowicz et al., 2007b). According to this reports, we assume an influence of the supraspinal TRPV1 receptors on the antiallodynia effects of WOBE437, rather than TRPV1 activation at the peripheral level. This hypothesis is in agreement with the present results, where AEA increases in the somatosensory cortex but decreases in plasma. It has been shown that subtle increments (1.2–1.5 times) in AEA levels in spinal cord or in PAG are enough to observe the antinociceptive or antiallodynia effects through TRPV1 activation (Maione et al., 2006; Starowicz et al., 2012). However, a proper measurement of AEA levels in the PAG and synovial tissue would be required to make any conclusions. Our data also show the involvement of CB2 receptors in the antiallodynic effect of WOBE437 which is fully prevented also by the selective CB2 receptor antagonist SR144528. Activation of CB2 receptor has been shown to counteract inflammatory pain, e.g., in models of neuropathic pain (Racz et al., 2008; Davis, 2014; Klauke et al., 2014). Interestingly, upon single administration p.o., WOBE437 induced a significant increase of 2-AG level in plasma. Since 2-AG is considered the endogenous full agonist at CB2 receptors (Gonsiorek et al., 2000; Soethoudt et al., 2017), we can speculate that the antiallodynic effect observed after single administration of WOBE437 reflects the pleiotropic biological actions of both ECs, specifically through the activation of TRPV1 (AEA) and CB2 receptors (2-AG). In the 3 days WOBE437 treatment we could not use the TRPV1 antagonist capsazepine because the mice showed an unexpected hypersensitivity, which appeared to be caused by concomitant WOBE437 and capsazepine administration. Our data suggest that the potentiation of EC signaling obtained by blocking cellular EC reuptake, and potentially interfering with AEA trafficking in a bidirectional manner (*vide infra*), leads to alleviation of allodynia by a common potentially synergistic signaling pathway activated by cannabinoid CB2 and CB1 receptors, PPARγ and possibly TRPV1. On the other hand, the antiinflammatory effect seems to be associated to more than one mechanism of action in which activation of CB2 receptors is predominant. Thus, EC signaling through CB1 receptor and PPARγ activation might only partially contribute to the antiinflammatory effect in this mouse model of monoarthritis. Since WOBE437 after 3 days treatment did not influence transcription of CB receptors or the enzymes involved in the generation of ECs we excluded indirect effects on gene expression of the major proteins of the ECS.

A possible common signaling node activated by cannabinoid CB1, CB2, and PPARγ receptors could be at the transcriptional level. In animal models of adjuvant-induced arthritis, osteoarthritis and neuroinflammation, it has been observed that independent activation of CB2 receptor and PPARγ can inhibit the activation of the canonical transcription factor NF-κB (Fakhfouri et al., 2012; Saravanan et al., 2014) and the mitogen-activated protein kinases (MAPKs) (Li et al., 2013; Moens et al., 2013; Ribeiro et al., 2013; Ma et al., 2015). This inhibition attenuates the expression and final release of pro-inflammatory mediators such tumor necrosis factor-α, interleukin 1-β, but also iNOS and COX2 (Fakhfouri et al., 2012; Luo et al., 2014), which are associated with the pain sensitization and the edema formation seen after CFA injection (Khan et al., 2013; Li et al., 2013; Luo et al., 2014). On the other hand, activation of cannabinoid CB1 receptors can also modulate NF-κB and MAPKs pathways via upregulation of the transcriptional activity of PPARγ (Du et al., 2011; Carroll et al., 2012; Fakhfouri et al., 2012; Ignatowska-Jankowska et al., 2014). In addition, ECs have been shown to modulate the activation of PPARγ through three possible mechanisms: (1) direct binding/activation, (2) via metabolites generated after AEA and 2-AG degradation, or (3) via cannabinoid CB1/CB2 receptor-mediated downstream signaling (i.e., MAPKs) (Burstein, 2005; O’Sullivan, 2007; O’Sullivan and Kendall, 2010). Therefore, ECs might modulate the inflammatory stage in monoarthritic mice through the inhibition of NF-κB/MAPKs signaling pathways, as a plausible explanation of the indirect polypharmacological effects of WOBE437 after 3 days of treatment. Nevertheless, further experiments are necessary to understand the exact molecular mechanism. There are not direct reports about the involvement of PPARγ in the pharmacological effects of MAGL or FAAH inhibitors *in vivo*. However, in an *in vitro* model of neuroinflammation, the pharmacological effects of the MAGL inhibitor JZL184 and the FAAH inhibitor URB597 were shown to be mediated by PPARγ in a CB1 receptor-depended manner (Du et al., 2011). In addition, the antidyskinetic effects of URB597 in combination with capsazepine were reversed by a non-selective PPAR antagonist, and in the same model a PPARγ selective agonist showed a significant reduction of dyskinesia (Martinez et al., 2015). Conversely, in an acute model of carrageenan-induced inflammatory hyperalgesia, PPARα but not PPARγ was involved in the analgesic effects of URB597 (Jhaveri et al., 2008). These reports might suggest that PPARγ is primarily involved in chronic but not acute inflammatory conditions (Villapol, 2018). Thus, cannabinoid receptors and PPARγ may potentially constitute a synergic antiinflammatory signaling network. The pharmacological differences between WOBE437 and FAAH/MAGL inhibitors could involve tissue selectivity, the degree of increment in ECs levels and the differential extracellular EC accumulation leading to increased cannabinoid CB1 and CB2 receptor activation. However, the present data point toward a more complex action of WOBE437 as also the intracellular AEA receptors TRPV1 and PPARγ were involved in the analgesic effects of this EC reuptake inhibitor, accompanied by a weak but significant decrease of AEA in plasma. Therefore, we cannot exclude bidirectional transport effects *in vivo* as shown previously *in vitro* (Chicca et al., 2012; Nicolussi and Gertsch, 2015).

## Conclusion

The novel EC reuptake inhibitor WOBE437 not only exhibits a high potency and selectivity (Chicca et al., 2017) but also matches the bioavailability criteria (in mice) that depict an adequate pharmacological tool compound. By applying WOBE437 in different mouse models of pain and inflammation, we found that the inhibition of EC reuptake triggers differential effects via elevation of ECs in different tissues and distinct pathophysiological/nociceptive contexts. This is in strong agreement with data obtained from the dual inhibition of AEA and 2-AG degradation (Anderson et al., 2014). Moreover, our data obtained with WOBE437 treatment reveal the noteworthy polypharmacology mediated by cannabinoid CB2, CB1, TRPV1, and PPARγ receptors in a mouse monoarthritis pain model. This indicates that this class of ECS modulators has the potential to exert therapeutic effects in chronic inflammatory conditions in which the pleiotropic effects of AEA and 2-AG counteract the pathophysiology. Therefore, SERIs together with non-selective FAAH/MAGL inhibitors are promising indirect cannabimimetics and should be further investigated in preclinical models of chronic inflammatory pain.

## Ethics Statement

This study was carried out in accordance with the recommendations of NOM-062-ZOO-1999 and Code of Ethics of the Directive 2010/63/EU. The protocol was approved by the Departamento de Farmacología, Centro Universitario de Ciencias Exactas e Ingenierías.

## Author Contributions

JV-P and JG designed the project. JV-P, JG, AC, MF-S, and IR-M planned and supervised the research. IR-M and MF-S carried out the experiments. IR-M obtained and processed the data. IR-M and JG drafted the manuscript. IR-M, JG, AC, and JV-P contributed to the writing of the manuscript. All authors saw the data, discussed the interpretation of experiments, and approved the final version.

## Conflict of Interest Statement

JG has been mentioned as an inventor in the patent WO2010136221A1. The other authors declare that the research was conducted in the absence of any commercial or financial relationships that could be construed as a potential conflict of interest.
